# Predicting Homebound Status in US Older Adults Using Data From the National Health and Aging Trends Study

**DOI:** 10.1093/geroni/igaf122.1030

**Published:** 2025-12-31

**Authors:** Anis Davoudi, Yifan Liu, Jennifer Schrack, Katherine Ornstein

**Affiliations:** Johns Hopkins University, Baltimore, Maryland, United States; Johns Hopkins University, Baltimore, Maryland, United States; Johns Hopkins Bloomberg School of Public Health, Baltimore, Maryland, United States; Johns Hopkins University, Baltimore, Maryland, United States

## Abstract

At least 5% of U.S. older adults are homebound —defined as primarily confined to their residence— a condition associated with adverse health outcomes and increased costs. Timely and accurate prediction of homebound status can ensure effective implementation of interventions to support aging in place and long-term care planning. While several risk factors have been identified for homeboundness (e.g., dementia, low income), there is limited work investigating prediction. We aggregated data from Round 1 and Round 5 of the National Health and Aging Trends Study (NHATS), a nationally representative panel study of U.S. Medicare beneficiaries aged ≥65 years, to predict homebound status. We included 43 sociodemographic, clinical, functional and environmental factors from the NHATS surveys as predictors. Homebound was defined as “Never/rarely leaves home”. We developed and evaluated CatBoost machine learning models for homebound prediction, using nested cross-validation for hyperparameter tuning and class weights to address outcome imbalance. Our final dataset included 8,651 not-homebound participants at baseline (age mean (SD): 76.6 (7.6) years, 56.2% female). 11.2% became homebound within the following four years). The final model achieved 0.75 accuracy, 0.75 specificity, 0.76 sensitivity, 0.28 precision, 0.82 AUC, and 0.41 F1-score. Short Physical Performance Battery (SPPB) score, driving, ability to walk 3 blocks, ADL dependency, and income quartile were the top 5 ranking features. These findings illustrate the feasibility of predicting homebound status among older adults, enabling focused interventions before homeboundness occurs. Moreover, identifying modifiable predictors enables the design of data-driven targeted interventions to enhance mobility and promote functional independence.

